# Microstructural Evolution, Mechanical Properties and Tribological Behavior of B_4_C-Reinforced Ti In Situ Composites Produced by Laser Powder Bed Fusion

**DOI:** 10.3390/ma16134890

**Published:** 2023-07-07

**Authors:** Jingguang Du, Yaojia Ren, Xinyan Liu, Feng Xu, Xiaoteng Wang, Runhua Zhou, Ian Baker, Hong Wu

**Affiliations:** 1 State Key Laboratory of Powder Metallurgy, Central South University, Changsha 410083, China; 2 Farsoon Technologies, Changsha 410205, China; 3 Research Institute of Smart Manufacturing, China Railway Construction Heavy Industry Co., Ltd., Changsha 410100, China; 4 School of Mechanical and Aerospace Engineering, Nanyang Technological University, Singapore 639798, Singapore; 5 Thayer School of Engineering, Dartmouth College, Hanover, NH 03755, USA

**Keywords:** laser powder bed fusion, titanium composite, microstructural evolution, mechanical property, tribological behavior

## Abstract

Based on the advantage of rapid net-shape fabrication, laser powder bed fusion (LPBF) is utilized to process B_4_C-reinforced Ti composites. The effect of volumetric energy density (*VED*) on the relative density, microstructural evolution, tensile properties and wear behaviors of B_4_C-reinforced Ti composites were systematically investigated. The LPBF-ed samples with high relative density (>99%) can be achieved, while the pores and un-melted powders can be observed in the sample owing to the low energy input (33 J/mm^3^). The additive particulates B_4_C were transformed into needle-like TiB whiskers with nano-scale while C dissolved in the Ti matrix. Fine-scale grains (<10 μm) with random crystallographic orientation can be achieved and the residual stress shows a downtrend as the *VED* increases. Through the analysis of the tensile and wear tests, the sample at 61 J/mm^3^ *VED* showed a good combination of strength and wear performance, with an ultimate tensile strength of 951 MPa and a wear rate of 3.91 × 10^−4^ mm^3^·N^−1^m^−1^. The microstructural evolution in *VED* changes and the corresponding underlying strengthening mechanisms of LPBF-ed Ti + B_4_C composites are conducted in detail.

## 1. Introduction

Titanium and its alloys have the advantages of high specific strength and stiffness combined with superior corrosion resistance, suitable for many industries including aerospace, marine, and automotive. However, the wear resistance of Ti limits the application of the areas mentioned above. To date, much attention has been devoted to titanium matrix composites (TMCs), which utilizes particulate reinforcement technology for strengthening the tribological properties, such as TiB, TiC, and TiN [1,2,3]. Nevertheless, the limited bond strength between ex situ category reinforcements and the Ti matrix may cause premature failure. As one of the ceramic particulates used, B_4_C shows superior stability combined with low density (2.52 g/cm^3^) and high hardness (~3000 HV) [4,5]. The introduction of B_4_C can efficiently reduce the weight of TMCs and the in situ formed TiB and TiC ceramic-reinforcement phase in TMCs leads to good interfacial bonding, outstanding thermodynamic stability, and fine-scale distribution [6,7,8,9]. In addition, the in situ reinforcement phase can provide an improvement in the mechanical properties of TMCs. For instance, Zhang et al. [10] demonstrated that the strength and high-temperature creep behavior of the Ti matrix increased with in situ TiB formation. Yu et al. found that fine in situ TiC particles can be obtained in TiC/Ti coatings processed by induction cladding, which is beneficial to harden the Ti matrix [11].

Many traditional manufacturing processes, such as vacuum casting or powder metallurgy, have been utilized to prepare Ti + B_4_C composites [12,13,14]. However, several issues, e.g., poor wettability, inferior densification response, and cracks, restrict the application of TMCs traditional processes. Besides, coarse carbide and boride particles are formed in TMCs through conventional processing methods owing to the low cooling rate after processing. These brittle phases are the origin of crack initiation and propagation [12,15].

As a promising 3D printing method, laser powder bed fusion (LPBF) can produce complex-shaped parts with almost fully dense structure, which enables to reduce the cost and improves the efficiency of the TMCs process [16,17,18,19]. During the LPBF process, a high laser power melts individual layers of material. Transient high temperature (~10^5^ K) and ultra-high cooling rate (10^4^~10^6^ K/s) can be obtained from a high beam scanning speed (500–1500 mm/s), leading to fine microstructures and superior mechanical properties [20,21,22,23]. These indicate that LPBF is likely to be an optimal process to produce TMCs with B_4_C addition.

Despite the benefits, LPBF fabrication of TMCs is limited by quality defects including pores and cracks due to its complex thermal history [24,25,26]. To obtain an in-depth understanding of the LPBF processing parameters–microstructure–properties relationship, manipulating the LPBF processing parameters, e.g., laser power and scanning speed, can be used to control the quality of LPBF processed components [27,28]. However, there are numerous LPBF processing parameters, which are interactive. To summarize, volumetric energy density (*VED*) has been used as an engineering parameter to predict the microstructure and properties of LPBF-ed alloys [29]. In the study of Wu et al. [30], the influence of *VED* on microstructural evolution and mechanical behaviors of LPBF-ed AlSi10Mg was investigated. They found that the relative density and yield strength of LPBF-ed AlSi10Mg decreased as *VED* increased from 40 to 90 J/mm^3^. Liu et al. systematically studied the effects of the *VED* on LPBF-ed Ti6Al4V and showed that the tensile strength increased and then dropped with increasing *VED* (32.7 to 132.4 J/mm^3^) [31]. This indicates that the effect of *VED* on different alloy systems is different. Currently, optimization of the LPBF processing parameters for Ti + B_4_C is rarely reported [32]. The influence of *VED* on the microstructure and properties of Ti + B_4_C composites needs further exploration. Thus, this paper aims to obtain an understanding of solidification microstructural evolution and the mechanical properties and tribological behavior of these composites.

In the present study, B_4_C-reinforced Ti in situ composites produced by LPBF were used as a model to investigate the effect of *VED* on the microstructural evolution, mechanical properties, and tribological behavior. As a parametric study, an optimal LPBF processing parameter was determined to produce Ti composites with high density and enhanced properties. In addition, the corresponding strengthening mechanisms of LPBF-ed Ti + B_4_C composites are studied.

## 2. Experiments

### 2.1. Powder Preparation

**Figure 1:**
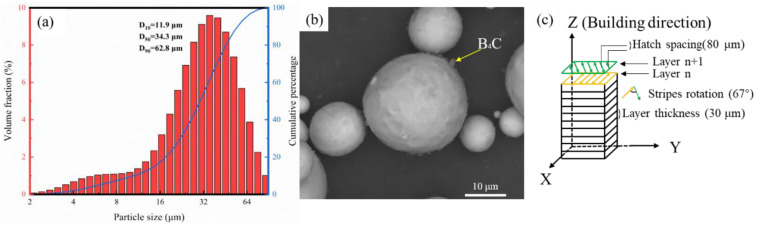
(**a**) Distribution of particle size for the Ti + B_4_C mixture, (**b**) SE image of the Ti + B_4_C mixture, and (**c**) schematic illustration of the laser scanning strategy.

Spherical unalloyed titanium powders (TA1, D10 = 18.4 μm, D50 = 30.6 μm, D90 = 47.1 μm, purchased from TIJO Inc., Changsha, China) and nano-sized B_4_C powders (TIJO Inc., Changsha, China) with an average diameter~500 nm was used in this study. The chemical composition of unalloyed Ti powder was detected using an inductively coupled plasma atomic emission spectrometer (ICP-AES), seen in Table 1. The powders were blended under argon in a mixer at room temperature for 12 h with a rotation rate of 20 rpm, and its distribution of particle size was seen in Figure 1a. The B_4_C content was 0.5 wt.%. A secondary electron (SE) image of the as-mixed powders is shown in Figure 1b.

### 2.2. LPBF Process

Both cubic (10 × 10 × 10 mm^3^) and dogbone-shaped tensile samples (gauge length 9.525 mm, gauge width 2 mm and thickness 2 mm) of the titanium-based composite were produced using an FS121 M LPBF machine equipped with a 500 W fiber laser (Farsoon Inc., Changsha, China). Several samples were built on Ti base plates with area dimensions of 110 mm × 110 mm. Stripes in subsequent layers were rotated by 67° with respect to the previous layer, and sample borders were outlined with a separate perimeter scan. A schematic of the laser scanning strategy is shown in Figure 1c. During the LPBF processing, high-purity argon was used to fill the processing chamber, resulting in less than 100 ppm oxygen present. The *VED* can be calculated using equation (1) from the LPBF parameters used, i.e., the laser power (P, 160–280 W), the scanning speed (*v*, 1000–2000 mm/s), the hatch distance (h, 80 μm), and the layer thickness (t, 30 μm).

(1) $$\mathrm{VED}=\frac{P\;}{v\mathrm{ht}}$$

### 2.3. Microstructural Characterization

For microstructural characterization, specimens were mirror polished using SiC paper and 0.25 μm SiO_2_ suspension successively, which are etched using Kroll’s reagent (2 mL HF, 6 mL HNO_3_, and 92 mL H_2_O) for ~10 s. Optical microscopy (OM, LEICA DM4500P, LEICA, Deerfield, IL, USA) was used to characterize the microstructure at low magnification. Phase identification was performed using an X-ray diffractometer (XRD, Advance D8, Bruker, Billerica, MA, USA) with Cu-Kα radiation and a step size of 0.02°. Further microstructural characterization was performed using a scanning electron microscope (SEM, MAIA3 TESCAN, Brno, Czechia) at an accelerating voltage of 20 kV. Texture analysis of the samples was performed using electron backscattered diffraction (EBSD) at a step size of 0.7 μm. Transmission electron microscope (TEM, FEI Titan G2, FEI, Hillsboro, OR, USA) analysis was performed at an accelerating voltage of 200 kV, and the compositions were determined using energy dispersive spectrometer (EDS).

### 2.4. Mechanical and Tribological Tests

**Figure 2:**
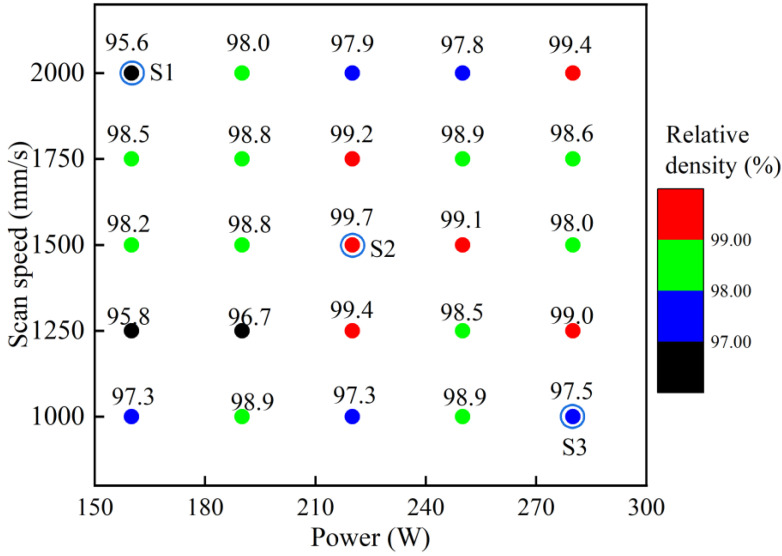
Relative density of LPBF-ed Ti + B_4_C composite.

The physical and mechanical properties of the LPBF-ed specimens were investigated via density measurements, nanoindentation measurements, tensile tests, and tribological studies. The density (*ρ*) was determined using the Archimedes method from:

(2) $$\rho =\frac{W_{a}\;\times \;\rho_{w}}{W_{a}\;-\;W}$$

where *W*_a_ and *W* are the weights of the sample in air and water, respectively, and $\rho_{w}$ is the density of the water. The density machine is Sartorius MSA324S-000-DU and each reported value is the average of three measurements. The theoretical density of Ti composite is 4.486 g/cm^3^. After analyzing the relative density results (Figure 2), the three specimens (named S1, S2, and S3) with *VED* values ranging from 33 to 117 J/mm^3^ are selected and discussed in this paper: the processing parameters associated with these specimens are listed in Table 2.

The nanoindentation measurements were undertaken using a MCT + UNHT machine (CSM Company, Kaisten, Switzerland) at room temperature. The load was set at 30 mN load with a loading time of 15 s and a Berkvoich indenter was used. Each specimen was measured at least 3 times. The reduced Young’s modulus is defined as:

(3) $$\frac{1}{E_{r}}=\frac{1\;-{\;v}^{2}}{E}+\frac{1\;-{\;v}_{i}^{2}}{E_{i}}$$

where *E* and *v* are the elastic modulus and Poisson’s ratio for the specimen, while *E*_i_ and *v*_i_ are the corresponding values for the indenter.

Tensile tests were performed at room temperature at an initial strain rate of 1 × 10^−3^s^−1^ on specimens cut using a wire electrical discharge machine perpendicular to the building direction and ground to a surface finish using 3000 grid SiC paper. The strain was measured with a video probe. Dry sliding wear tests were performed on specimens’ flat ground. A 4 mm diameter Si_3_Ni_4_ ball was used as the counter-material. The load, rotation speed, rotation radius, and time were at 10 N, 10 Hz, 1 mm, and 30 min, respectively. The tensile fracture surfaces and worn surfaces were examined using the secondary electron mode in the SEM.

## 3. Results

### 3.1. Microstructure

**Figure 3:**
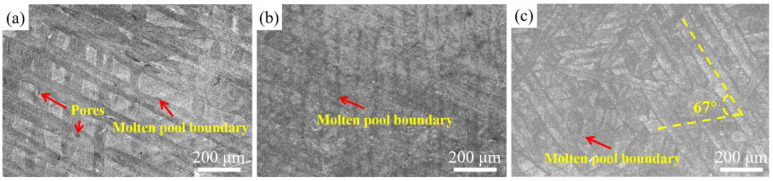
OM images of (**a**) S1, (**b**) S2, and (**c**) S3.

Figure 3 shows typical OM images of S1, S2 and S3 viewed in the x–y plane. The melt track, also known as the melt pool, can be clearly observed. It is evident that the rotation angle between melt tracks is about 67°, as expected. Micropores were presented in S1 resulting in lower relative density (~96%), which may be attributed to insufficient energy penetration at the lowest *VED*. The morphology of S2 was similar to that of S3. The excellent track-to-track bonding suggested that the alloys with a near-dense structure show good mechanical properties.

**Figure 4:**
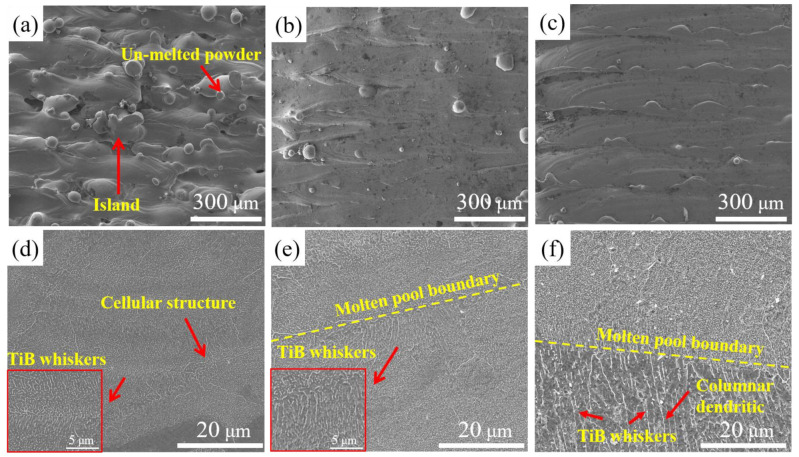
SE images of surface and microstructure of LPBF-ed Ti + B_4_C in situ composites: (**a**,**d**) S1; (**b**,**e**) S2; (**c**,**f**) S3.

Figure 4 shows the surface morphologies of S1, S2 and S3. There were both un-melted powders and island-shape phenomenon on the surface of S1 (Figure 4a), arising from the limited energy penetration at a *VED* of 33 J/mm^3^. As the *VED* increased to 61 J/mm^3^ (specimen S2), both the number of un-melted powders and the surface island-shape phenomenon decreased substantially. When the *VED* further increased to 117 J/mm^3^ (specimen S3), the surface became smoother and free of any evident defects, a result that can be attributed to the greater laser power penetrating the powder-bed [33].

Figure 4d–f show typical microstructures of LPBF-ed samples in the x–y plane. The *VED* exerts a significant influence on the microstructure of the Ti + B_4_C composites. Unlike the other specimens, S1 exhibited cellular microstructure. As the *VED* increased from 61 J/mm^3^ to 117 J/mm^3^, a change to a columnar structure can be observed. The difference in microstructure is dependent on the complex solidification behaviors in the molten pool caused by various *VED*, which is discussed in Section 4.1. In addition, needle-like TiB whiskers of different sizes can be seen in all three samples. Clusters composed of TiB whiskers were present in the LPBF-ed microstructure, where parallel whiskers were stuck to each other. The TiB whiskers grew into this morphology due to the faster growth rate in the [010] axis than that in other directions [34].

### 3.2. EBSD Characterization

**Figure 5:**
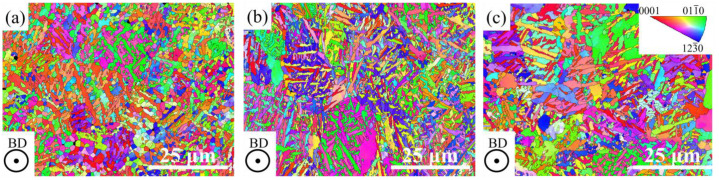
IPFs perpendicular to BD of samples with different *VED*: (**a**) S1; (**b**) S2; (**c**) S3.

The effect of *VED* on the grain size and crystallographic orientations of the LPBF-ed B_4_C-reinforced Ti in situ composites was studied using EBSD. Inverse pole figures (IPF) viewed perpendicular to the building direction (BD) of S1, S2, and S3 are shown in Figure 5. The grains in the three samples were mostly smaller than 10 μm, while a few more coarse grains were observed in S3. The average α lath width of three samples was determined to be 0.67 μm, 0.75 μm, and 0.89 μm, respectively. The small increase in lath width can be attributed to the greater heat input from the increased *VED*. Besides, the top-viewed crystallographic orientations of three specimens in Figure 5 show crystallographic directions were random.

### 3.3. TEM Analysis

**Figure 6:**
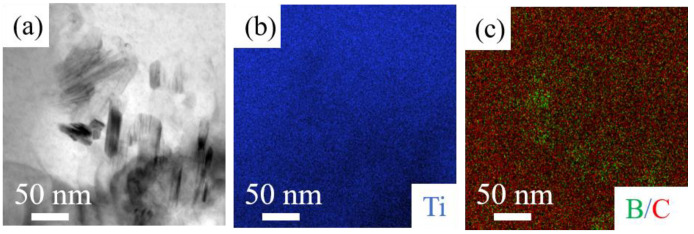
(**a**) BFTEM images of LPBF-ed S2; (**b**,**c**) corresponding EDS map showing the Ti and B/C distribution.

To further clarify the microstructure of the LPBF-ed Ti + B_4_C composites, specimen S2 was examined in the TEM. Figure 6a shows that the needle-like whiskers with sizes ranging from 50 to 100 nm were randomly distributed in the matrix. However, TiC and TiB are hardly observed in TEM-EDS maps. In α-Ti alloys, the solid solubility of C is 0.458 wt.% at 1173K and drops to 0.126 wt.% at 873K [35]. During the LPBF process, 0.104 wt.% of C dissolved into S2, where the carbon content is below the maximum carbon solubility. This is the reason that TiC particles are not observed at room temperature.

**Figure 7:**
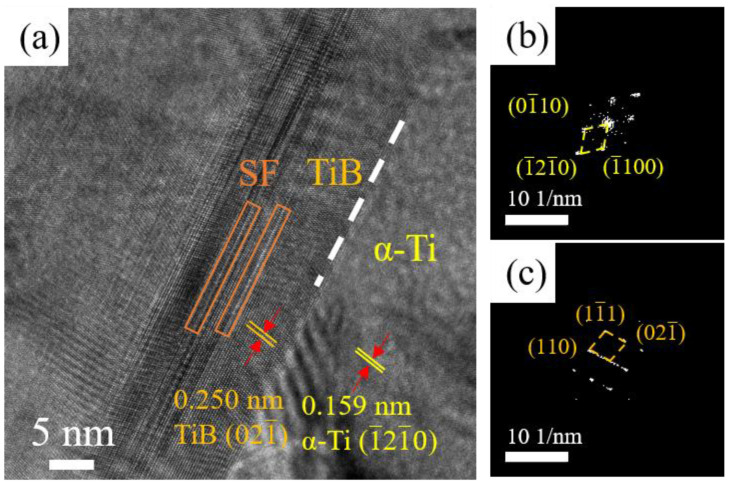
(**a**) HRTEM images of TiB whisker and the Ti matrix; (**b**,**c**) FFT patterns of α-Ti and TiB whisker, respectively.

High-resolution TEM (HRTEM) was performed to examine the interface between the TiB whisker and the α-Ti matrix. Figure 7a shows a clean interface between the matrix and the reinforcing phase, illustrating good bonding between the TiB whisker and the α-Ti. The α-Ti matrix and TiB whiskers were identified via determining the lattice parameters to be 0.159 nm and 0.250 nm, respectively. However, the theoretical lattice spacing of α-Ti_d{110}_ is known to be 0.147 nm, which is smaller than the experimental results. Kværndrup et al. reported that the interstitial atoms (O, N, or H) dissolution into α-Ti leads to the increase of lattice parameter [36]. The results indicated that C is dissolved into Ti, leading to lattice spacing expansion. The corresponding fast Fourier transform (FFT) patterns of the LPBF-ed Ti + B_4_C composite are shown in Figure 7b,c, which further confirms the existence of the TiB whisker and the α-Ti phase. In addition, intense streaking in the direction of TiB (110) was observed, demonstrating the existence of stacking faults (SF) in the TiB (110) plane. Kooi et al. reported a similar structure in laser-clad Ti-TiB [34].

### 3.4. Phase Identification

**Figure 8:**
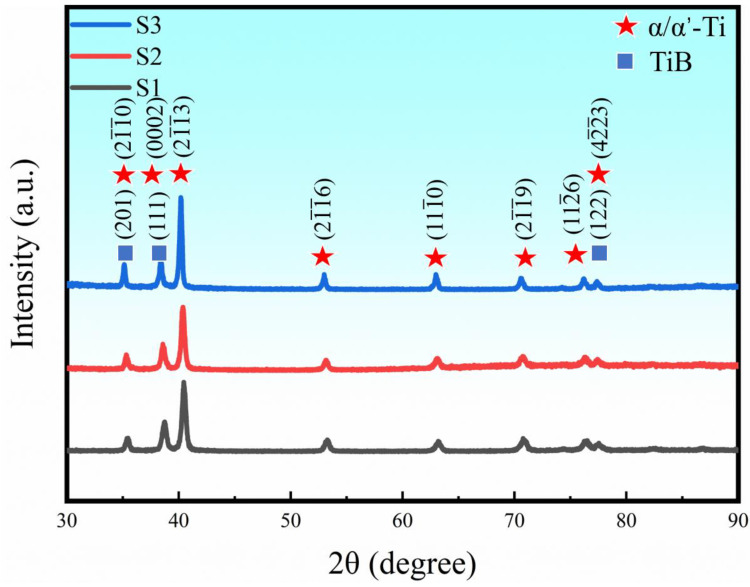
XRD results of three LPBF-ed samples with various *VED*s.

XRD patterns from S1, S2 and S3 are shown in Figure 8. Strong diffraction peaks from hexagonal Ti (HCP) and weak diffraction peaks from the small volume fraction of TiB were found. According to the Ti-B-C ternary phase diagram [37], an in situ reaction occurred during the solidification processing of LPBF-ed samples:

(4) $${\mathrm{Ti}+B}_{4}C\;\to \;\mathrm{\beta -Ti}+\mathrm{TiB}\;\to \;\mathrm{\alpha -Ti}+\mathrm{TiB}$$

### 3.5. Mechanical Properties

**Figure 9:**
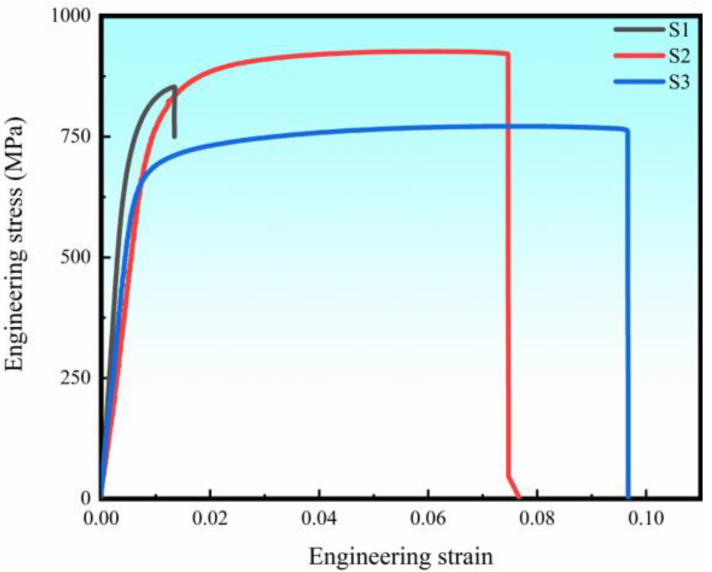
Engineering strain-stress curves of LPBF-ed B_4_C-reinforced Ti in situ composites.

Tensile tests were conducted to evaluate the LPBF-ed B_4_C-reinforced Ti in situ composites and their representative engineering stress-engineering strain responses are displayed in Figure 9: values for the yield strength (YS), ultimate tensile strength (UTS), elongation (ε) is summarized in Table 3. S1 had the lowest relative density (95.6%) and exhibited the lowest ε, but had the highest YS at 768 ± 10 MPa. Compared with S1, the YS value of S2 was ~30 MPa less at 738 ± 6 MPa, but its ε increased from 1.7 ± 0.4% to 6.3 ± 1.1%, presumably due to a lower number of defects. Compared with S2, S3 had the similar ε at 7.4 ± 1.9%. However, S3 had a lower YS value (664 ± 9 MPa) than those of the other two specimens. As increasing *VED*, the pore morphology in Ti alloys changed from irregular shape to near-spherical shapes [31]. During tensile test, samples with spherical pores have a more uniform distribution of strain, which may lead to better ductility performance than that of samples with irregular pores. The decrease of YS can be attributed to the coarse grain in S3 caused by large energy input. As shown in Table 3, the introduction of B_4_C to pure Ti can achieve high tensile strength and reasonable elongation with comparison of pure Ti produced by LPBF, wrought or cast [38,39,40,41,42].

**Figure 10:**
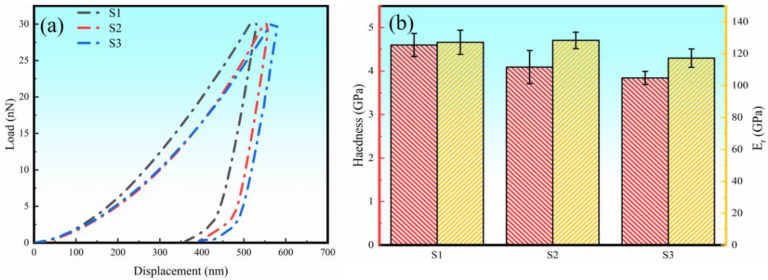
(**a**) Displacement-load response and (**b**) hardness and reduced Young’s modulus results of three samples.

Nanoindentation tests were conducted on the three specimens and the smooth load-displacement curves obtained are shown in Figure 10a. It can be observed that the indentation depth of the LPBF-ed Ti + B_4_C composites becomes slightly deeper with increasing *VED*. Figure 10b is a histogram of the hardness (*H*) and reduced Young’s modulus, (*E*r) for the three samples. The increasing *VED* led to a slight decrease in *H* from 4.6 GPa to 3.8 GPa. The reduced Young’s moduli of S1 and S2 were similar (~128 GPa) but were ~8% greater than that of S3.

### 3.6. Tribological Behavior

**Figure 11:**
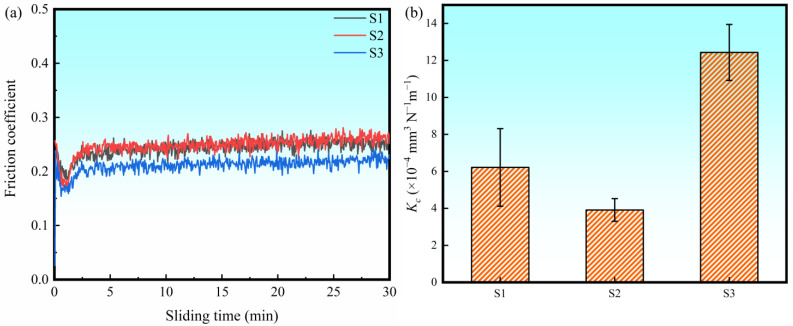
(**a**) Friction coefficient versus time, and (**b**) *K_c_* for the three samples.

Figure 11 presents the friction coefficient and wear rate from wear tests of the LPBF-ed Ti + B_4_C composites. The wear rate coefficient (*K_c_*) was calculated from (5):

(5) $$K_{c}=\frac{2\pi \mathrm{rA}}{\mathrm{FS}}\;$$

where r and A are the radius and area of wear tracks, respectively, and F and S are the load and sliding distance, respectively. The friction coefficient curves of the three samples experienced a short fluctuation within an unstable period (about 3 min) and then became more stable. The average friction coefficients of S1, S2, and S3 were calculated during the steady period (3-30 min). S1 exhibited both a high friction coefficient (0.25 ± 0.01) and a high *K_c_* (6.2 ± 2.1 × 10^−4^ mm^3^·N^−1^m^−1^). A similar friction coefficient (0.25 ± 0.01), but the lowest *K_c_* (3.9 ± 0.6 × 10^−4^ mm^3^·N^−1^m^−1^) was obtained for S2. Interestingly, S3 exhibited the lowest friction coefficient (0.22 ± 0.01) but the highest *K_c_* (12.4 ± 1.5 mm^3^·N^−1^m^−1^).

**Figure 12:**
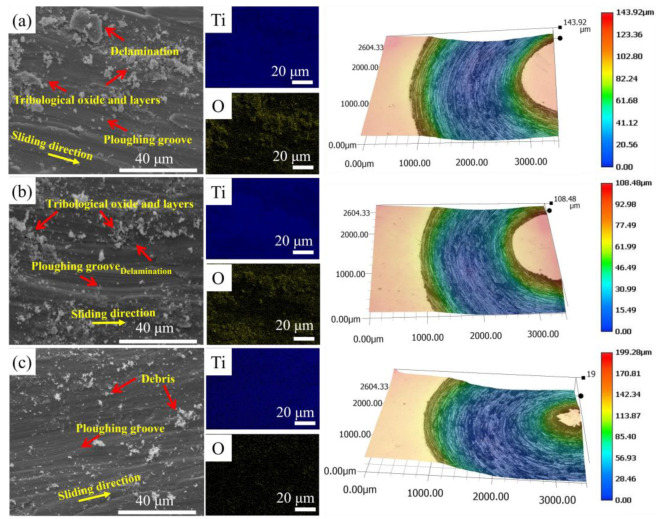
SE images of worn surfaces, corresponding EDS maps and 3D surface profiles: (**a**) S1, (**b**) S2, and (**c**) S3.

The worn surfaces (typical morphologies and 3D surface profiles) and corresponding EDS mapping of S1, S2, and S3 are presented in Figure 12. The shallower grooves along the sliding distance on S1 and S2 surfaces were clear while the severer grooved scratches and more debris can be observed on the S3 surface. Based on the EDS mapping result, it was demonstrated that wear debris were tribological oxides and the tribological layers were composed of oxides. In addition, white debris detected in Figure 12 were considered an oxidative wear character because of the exposure to the air during the dry sliding wear test [43,44].

## 4. Discussion

### 4.1. Microstructural Evolution

When the laser penetrates the powder-bed, the Ti powders first melted and then reacted with the B_4_C particles in the molten pool. However, inadequate penetration led to un-melted powder and island-shape phenomenon, which produced a rough surface, as observed in specimen S1. With increasing *VED*, the composite powders could be fully melted and the surface became smooth. In general, the viscosity of the fluid is strongly determined by the temperature in the molten pool, which has a significant influence on surface quality. According to Takamichi [45], the viscosity of fluid (*μ*) in the molten pool can be calculated from (6):

(6) $$\mu =\alpha \sqrt{\frac{m}{kT}}\gamma$$

where *α* is a constant, *m* is the atomic mass, *k* is the Boltzmann constant, *γ* is the surface tension of fluid and *T* is fluid temperature. The surface tension of fluid dropped with increasing fluid temperature [46]. Therefore, the viscosity of fluid decreased caused by a higher *VED*. The fluid in the molten pool spreads easily and a high-quality surface without defects was obtained for higher *VEDs*.

Characteristic solidification microstructures in Figure 4 were formed in the LPBF process. When the laser interacted with the powder bed, B_4_C is prone to react with Ti and transforms into in situ formed TiB whiskers. TiB whiskers are melted into the liquid over its melting point (2473 K) or partially dissolved in the region below 2473 K. During the solidification process, the solute atoms were ejected at the solid/liquid interface front, which led to a higher concentration of solute. The retained TiB whisker was captured to act as grain boundaries and eutectic TiB whiskers solidified between the Ti grains. Besides, the length of TiB whiskers became larger, which may be caused by longer solidification time as *VED* increased. The solidification morphology is determined by the ratio of the temperature gradient (G) to solidification rate (R) while G×R governs the microstructural scale. G is given by the temperature field caused by the laser and R is related to beam velocity and the melt pool shape. A change from cellular to columnar structures occurred due to differences in G and R in the molten pool caused by different *VEDs*. The metastable cell structure of S1 formed in the LPBF process can be attributed to the moderate G/R. At the higher *VED* in S3, a lower G/R was obtained, which led to columnar dendrites [47]. Therefore, it was seen that increasing *VED* may lead to a reduced G/R, which changes the microstructure.

### 4.2. Mechanical Properties Analysis

The increment in the strength of LPBF-ed Ti + B_4_C in situ composites can be attributed to three strengthening mechanisms: (1) grain refinement caused by the heterogeneous nucleation on TiB; (2) TiB whiskers acting as reinforcements; and (3) solid-solution strengthening from dissolved carbon. For a better understanding of the strengthening mechanisms, S2 was chosen to quantitatively estimate these effects.

Upon the addition of the B_4_C powders, the grain size decreased compared to LPBF-ed Ti [41]. Based on the Hall–Petch relationship, the increase in yield strength by grain size reduction is expressed as:

(7) $$\;\Delta \sigma_{\mathrm{H-P}}=K\left(d_{1}^{-\frac{1}{2}}{\;-\;d}_{2}^{-\frac{1}{2}}\right)$$

where *K* is a constant as 328 MPa μm^1/2^ [48], *d*_1_ and *d*_2_ are α-Ti grain size of the Ti + B_4_C composite and the titanium matrix, respectively.

Because of the limited solid solubility in the matrix (~0.02 wt.%), most of the boron was presented in the TiB whiskers. The fiber strengthening effect from the TiB whiskers can be calculated from:

(8) $$\;\Delta \sigma_{\mathrm{TiB}}=0{.5\sigma }_{\mathrm{YSm}}V_{\mathrm{TiB}}\frac{l}{d}\omega_{0}$$

where *σ*_YSm_ is the yield strength of the Ti matrix, *V*_TiB_, *l*/*d*, and ω_0_ are the volume fraction, aspect ratio, and whisker orientation factor for the TiB whiskers, respectively. The orientation of TiB whiskers is random and hence ω_0_ = 0.27 [49,50,51].

The solid solution strengthening from carbon in the Ti matrix can be expressed as [49]:

(9) $$\Delta \sigma_{S}=\frac{1}{\sqrt{3}}m_{T}\frac{1}{2\left(1+\upsilon \right)}E_{r}\eta^{\frac{3}{2}}{\;c}^{\frac{1}{2}}$$

where *m*_T_, *υ*, *E*_r_ are the Taylor factor, Poisson׳s ratio, and elastic modulus of the Ti alloy, respectively. *η* is related to the change in the lattice constants with carbon concentration in the Ti matrix [52] and *c* is the atom fraction of carbon (~0.43 wt.%).

Therefore, the theoretical YS of S2 can be calculated from:

(10) $$\sigma_{\mathrm{YS}}=\sigma_{\mathrm{YSm}}+\Delta \sigma_{\mathrm{H-P}}+\Delta \sigma_{\mathrm{TiB}}+\Delta \sigma_{S}$$

Using the Equations (7)–(10), the YS of S2 was calculated to be 698 MPa with parameters listed in Table 4, which agreed relatively well with the experimental value of 712 MPa. Notably, ∆$\sigma_{\mathrm{H-P}}$, $\Delta \sigma_{\mathrm{TiB}}$, and $\Delta \sigma_{S}$ are 134 MPa, 22 MPa, and 136 MPa, respectively. The largest increase in strength was from solution strengthening, accounting for 48%. Note that the tensile strength of S3 was significantly lower than that of others, which may be attributed to higher energy penetration resulting in coarser grains.

**Figure 13:**
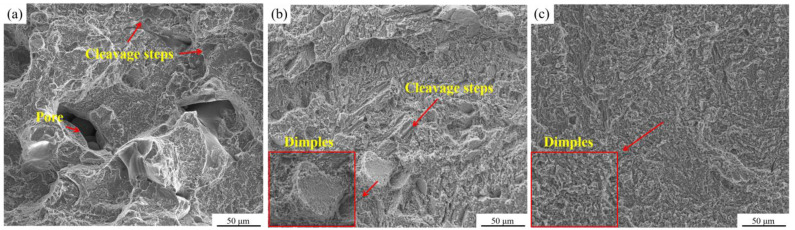
SE images of the fracture surfaces of (**a**) S1, (**b**) S2, and (**c**) S3.

Secondary electron images of the fracture surfaces of the LPBF-ed Ti + B_4_C composites are shown in Figure 13. There were several pores and cleavage steps evident in S1 caused by insufficient *VED* (Figure 13a). During tensile testing, crack propagation may begin at such defects, leading to worse ductile performance [54,55]. Such pores were not evident for S2 and S3, which explained the higher ductility for these two specimens. A combination of cleavage steps and dimples can be observed in Figure 13b, which demonstrated that the fracture mechanism was a mixture of brittle fracture and plastic deformation. However, the dimples were small and shallow, compared with that in Figure 13c. This phenomenon explained the plastic deformation in S2 was limited. The main fracture mechanism of S3 was plastic deformation because of the full dimples. In summary, the mechanism of fracture changed from brittleness to ductile failure with the enhanced *VED*.

### 4.3. Tribological Behavior Analysis

Different wear mechanisms of the LPBF-ed samples led to different tribological behavior. The shallow grooves along the sliding distance and tribological layers of S1 and S2 indicated that adhesive and abrasive wear mechanisms predominate during the wear test. The dominant wear mechanism of S3 was abrasive wear, demonstrated by severer grooved scratches and more white debris. The contact and relative sliding between materials and Si_3_N_4_ generated high tribological heat and local pressure, leading to the formation of tribological oxides and layers on the composite surface. During a long period of high-stress contact, cracks and delamination occurred on the tribological layers. In S1 and S2, tribological layers composed of wear debris were formed due to the applied stress during the wear test. Layers composed of tribological oxide existing on the sample surface can efficiently reduce wear rate and improve wear resistance [44]. In addition, the hardness and strength of materials were considered important indices judging wear resistance [56]. It can be inferred that tribological layers composed of oxide and better strength of S1 and S2 led to better tribological performance than that of S3 (12.4 ± 1.5 mm^3^·N^−1^m^−1^). While the lower relative density of S1 led to an unstable tribological performance (6.2 ± 2.1×10^−4^ mm^3^·N^−1^m^−1^), which may be due to un-melted powders and pores acting as crack initiation [57].

## 5. Conclusions

In situ Ti composites reinforced by B_4_C were successfully processed by LPBF. The effect of *VED* on relative density, microstructure, tensile properties, and wear behaviors have been investigated. The following conclusions are:

(1) A relative density higher than 99% was achieved in S2 and S3. The island-shape phenomenon and un-melted powders in S1 can be attributed to the lower viscosity of fluid caused by inadequate lower energy input.

(2) The microstructural evolution was influenced by *VED*. The cellular microstructure changes gradually to columnar morphology with *VED* increment. A small increase of grain size due to more heat input by *VED* and the crystallographic orientations are random

(3) A superior UTS of 951 ± 21 MPa combined with reasonable ductility (6.3 ± 1.1%) was achieved in the S2. The improved strength was attributed to grain refinement strengthening caused by TiB whiskers, TiB reinforcement mechanism, and solution strengthening of carbon in the Ti matrix. The fracture mechanism changed gradually from brittleness to ductile failure with the *VED* increases.

(4) A low wear rate and friction coefficient of S2 were reached 3.91 × 10^−4^ mm^3^·N^−1^m^−1^ and 0.252, respectively. Owing to tribological layers on the sample surface and better strength, S1 and S2 had a better wear behavior than that of S3.

## Figures and Tables

**Table 1:** The chemical composition of unalloyed Ti powder.

Unalloyed Ti Powder	H	C	N	O	Ti
Chemical composition (wt.%)	0.006	0.007	0.023	0.098	Bal.

**Table 2:** LPBF processing parameters for Ti + B_4_C composite specimens.

Specimen	P (W)	*v* (mm/s)	h (μm)	t (μm)	*VED* (J/mm^3^)
S1	160	2000	30	80	33
S2	220	1500	30	80	61
S3	280	1000	30	80	117

**Table 3:** Summary of the relative density and average mechanical properties.

Sample	Condition	YS (MPa)	UTS (MPa)	ε (%)	Reference
S1	LPBF	768	821	1.7	This work
S2	LPBF	738	951	6.3	This work
S3	LPBF	664	771	7.4	This work
Ti	LPBF	521	607	10.4	[38]
Ti	LPBF	590	665	19	[39]
Ti	LPBF	407	469	14.7	[40]
Ti	LPBF	420	510	18	[41]
Ti	Wrought	317	481	28.9	[38]
Ti	Cast	351	466	30	[42]

**Table 4:** Parameters value for calculation.

Parameter	Value	Reference
*K*	328 MPa μm^1/2^	[48]
*d*_1_	0.75 μm	Measured
*d*_2_	1.8 μm	[41]
$\sigma_{\mathrm{YSm}}$	420 MPa	[41]
*l*/*d*	20	Measured
*m*_T_	3.16	Measured
ω_0_	0.27	[53]
*E*_r_	128 GPa	Measured
η	0.08	[49]
*υ*	0.27	[49]

## Data Availability

The data presented in this study are available on request from the corresponding author.
